# Surface Morphology at the Microscopic Scale, Swelling/Deswelling, and the Magnetic Properties of PNIPAM/CMC and PNIPAM/CMC/Fe_3_O_4_ Hydrogels

**DOI:** 10.3390/gels2040030

**Published:** 2016-12-13

**Authors:** Marianna Uva, Andrea Atrei

**Affiliations:** 1 Dipartimento di Biotecnologie, Chimica e Farmacia, Università di Siena, Via A. Moro 2, 53100 Siena, Italy; marianna.uva@unisi.it; 2 CRISMA, Via. Matteotti 15 Colle di Val d’Elsa, 53034 Siena, Italy

**Keywords:** hydrogels, thermoresponsive materials, magnetic nanoparticles, poly(*N*-isopropylacrylamide) (PNIPAM), field emission scanning electron microscopy (FESEM)

## Abstract

Poly(*N*-isopropylacrylamide) (PNIPAM) hydrogels containing carboxymethylcellulose (CMC) and CMC/Fe_3_O_4_ nanoparticles were prepared. Free-radical polymerization with BIS as cross-linker was used to synthesize the hydrogels. The morphology at the microscopic scale of these materials was investigated using field emission scanning electron microscopy (FESEM). The images show that CMC in the PNIPAM hydrogels induces the formation of a honeycomb structure. This surface morphology was not observed for pure PNIPAM hydrogels prepared under similar conditions. The equilibrium swelling degree of the PNIPAM/CMC hydrogels (5200%) is much larger than that of the pure PNIPAM hydrogels (2500%). The water retention of PNIPAM/CMC hydrogels above the volume phase transition temperature is strongly reduced compared to that of pure PNIPAM hydrogel. Both PNIPAM/Fe_3_O_4_ and PNIPAM/CMC/Fe_3_O_4_ hydrogels exhibit a superparamagnetic behavior, but the blocking temperature (104 K) of the former is higher than that of the latter (83 K).

## 1. Introduction

Poly(*N*-isopropylacrylamide) (PNIPAM) hydrogels are the most investigated thermoresponsive hydrogels [1]. Because of their capability to change volume reversibly at a transition temperature, hydrogels of PNIPAM or co-polymer of PNIPAM have applications in several fields ranging from drug delivery to environmental remediation [2,3]. The introduction of magnetic nanoparticles (NPs) into PNIPAM hydrogels allows to heat remotely the hydrogels by applying alternating magnetic fields of suitable frequencies [2]. In most of these applications the rate of the deswelling/swelling processes across the volume phase transition temperature (VPTT) is important [4]. Unfortunately, the response of macroscopic PNIPAM hydrogels to temperature changes is rather slow [5,6,7]. The collapse of the swollen hydrogel (with expulsion of water molecules out of the polymer matrix) has a complex dynamics involving diffusion of polymer segments and conformation changes of the polymer chains [8]. A contribution to slow the thermal response of PNIPAM hydrogels comes from the so-called “skin effect”, which is the formation of a dense, vitrous layer which prevents the exchange of water molecules between the hydrogel and the surrounding [9]. Several methods have been introduced to obtain PNIPAM hydrogels featuring a faster response to temperature variations. Some of these methods use the copolymerization of NIPAM with hydrophilic molecules to eliminate the skin effect and form hydrophilic channels for the diffusion of water molecules [9,10]. Other methods are based on promoting the formation of interconnected pores or use PNIPAM microgels embedded in a macrogel [11,12]. It has been shown that the interpenetrating polymer network (IPN) or semi-IPN hydrogels of NIPAM with a hydrophilic polysaccharide such as sodium alginate have a faster response to temperature variation [13]. In the present work, we prepared PNIPAM hydrogels containing carboxymethylcellulose (CMC) and magnetite (Fe_3_O_4_) NPs coated with CMC. CMC, a hydrophilic, biocompatible derivative of cellulose, appears to be a suitable candidate to prepare PNIPAM hydrogels exhibiting a faster thermal response. The choice of CMC was also motivated by its ability to stabilize aqueous dispersions of magnetite NPs [14]. One of the aims of this study was to investigate the effect of CMC and CMC/Fe_3_O_4_ NPs on the morphology at micrometric scale and on the swelling/deswelling properties of PNIPAM hydrogels. The other aim was to study the effect of coating magnetite NPs with CMC on the magnetic properties of PNIPAM hydrogels containing Fe_3_O_4_ NPs. The morphology of the PNIPAM hydrogels was investigated by means of field emission scanning electron microscopy (FESEM). The magnetic properties of PNIPAM/Fe_3_O_4_ and PNIPAM/CMC/Fe_3_O_4_ hydrogels were studied by measuring magnetization versus magnetic field curves at various temperatures, and zero field cooling (ZFC) and field cooling (FC) curves. 

## 2. Results and Discussion

**Figure 1:**
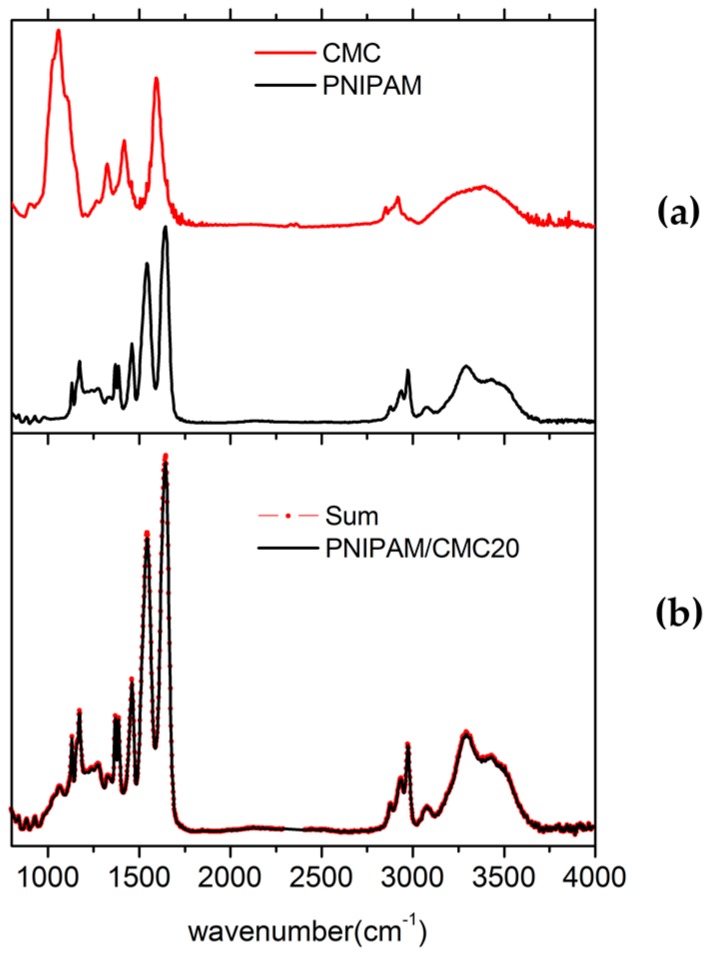
(**a**) Attenuated total reflectance–Fourier transform infrared spectroscopy (ATR-FTIR) spectra of carboxymethylcellulose (CMC) sodium salt and poly(*N*-isopropylacrylamide) (PNIPAM) hydrogel. The spectra were measured under the same instrumental conditions. (**b**) Fitting of the spectrum of the PNIPAM/CMC20 sample as a sum of the spectra of CMC and PNIPAM hydrogels weighted by optimized scaling factors. The spectra were measured for the lyophilized hydrogels.

The attenuated total reflectance–Fourier transform infrared spectroscopy (ATR-FTIR) spectrum measured for the PNIPAM hydrogel containing 20 wt % of CMC (PNIPAM/CMC20) can be fitted by summing the spectra of PNIPAM dry hydrogel and CMC. The best fit is obtained by optimizing the weight factors of the intensities of the spectra of PNIPAM hydrogel and CMC (Figure 1). This result suggests that there are no large interactions between the PNIPAM network and the CMC chains which, otherwise, would produce variations of the peak positions and widths in the spectrum of PNIPAM/CMC hydrogel with respect to those of the pure components. Because of partial overlapping of the main peaks of PNIPAM and CMC and the relatively small contribution of CMC to the intensity of the PNIPAM/CMC20 spectrum, Fourier transform infrared spectroscopy (FTIR) is not able to reveal subtle changes in the spectrum, resulting from possible interactions between PNIPAM and CMC or cross-linking of CMC chains. Hence, attenuated total reflectance (ATR)-FTIR results are consistent with the formation of a semi-IPN, but the cross-linking of CMC chains, leading to a full IPN [15], cannot be ruled out. 

**Figure 2:**
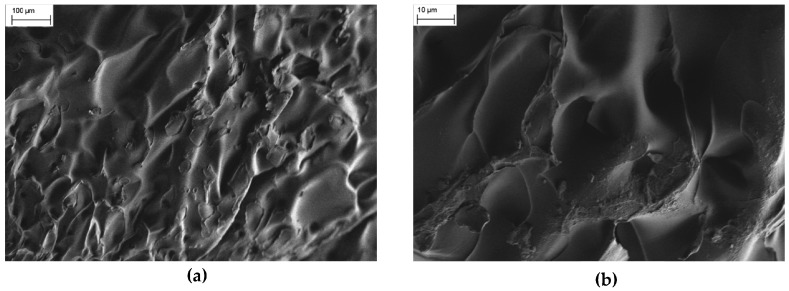
(**a**) Field emission scanning electron microscopy (FESEM) image collected for a pure PNIPAM hydrogels. (**b**) FESEM image collected for a pure PNIPAM hydrogel at a larger magnification.

FESEM images show that the microstructures of pure PNIPAM and PNIPAM/CMC lyophilized hydrogels are significantly different. Pure PNIPAM hydrogels have a morphology consisting of relatively flat foils over which large craters (size around 100 nm) are visible (Figure 2a). Even at higher magnifications, PNIPAM hydrogels exhibit a non-porous structure (Figure 2b). The images shown here are representative of the morphology of the whole samples.

**Figure 3:**
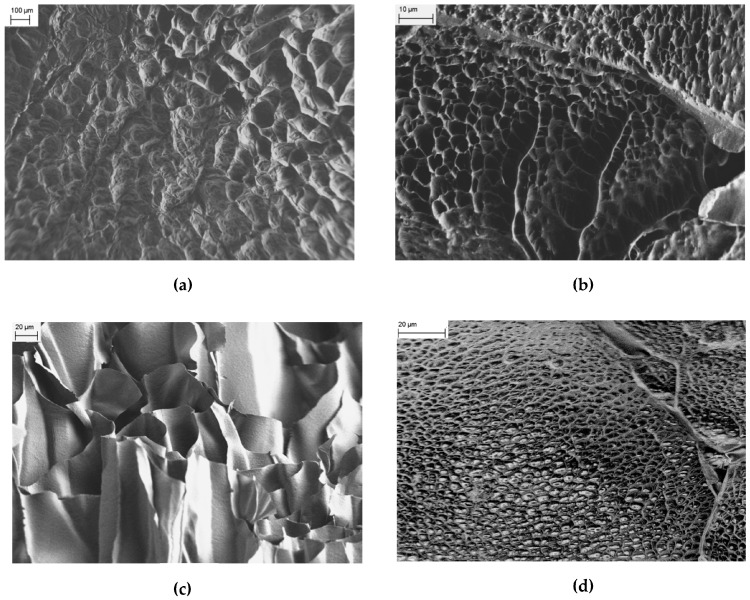
(**a**) FESEM image collected for a PNIPAM/CMC20 sample. (**b**) FESEM image collected for a PNIPAM/CMC20 sample at a larger magnification. (**c**) FESEM image of an inner cross section of the PNIPAM/CMC20 sample. (**d**) FESEM image of a PNIPAM hydrogel containing 5 wt % of CMC.

On the other hand, FESEM images of PNIPAM/CMC are characterized by a honeycomb-like structure (Figure 3a,b), with features smaller than those observed in the images of pure PNIPAM. Images of internal cross sections of the PNIPAM/CMC hydrogels show that a network of interconnected pores exists inside the hydrogels. The observed honeycomb-like structure is probably due to the emergence of micropores at the surface of the PNIPAM/CMC hydrogels as suggested by the images measured for the inner cross-sections of the hydrogel (Figure 3c). The surface morphology of the PNIPAM/CMC hydrogels does not change significantly in the explored range of CMC concentration (that is from 5 wt % to 20 wt %) as far as the presence of pores is concerned. However, the size of the micropores tends to be smaller for the lower CMC concentration (Figure 3d).

**Figure 4:**
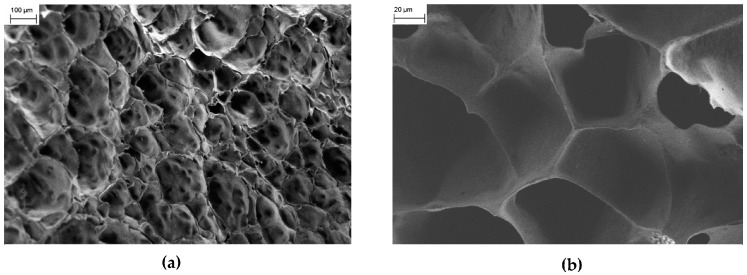
(**a**) FESEM image of the PNIPAM/CMC5/Fe_3_O_4_ hydrogel. (**b**) FESEM image collected for the NIPAM/CMC5/Fe_3_O_4_ hydrogel at a larger magnification. FESEM image of a PNIPAM/CMC sample containing 5 wt % of CMC.

The surface morphology of the hydrogels is not significantly affected by the presence of CMC/Fe_3_O_4_ NPs as shown by the FESEM images of the PNIPAM/CMC hydrogel containing 4.3 wt % magnetite (PNIPAM/CMC5/Fe_3_O_4_) samples (Figure 4a,b).

**Figure 5:**
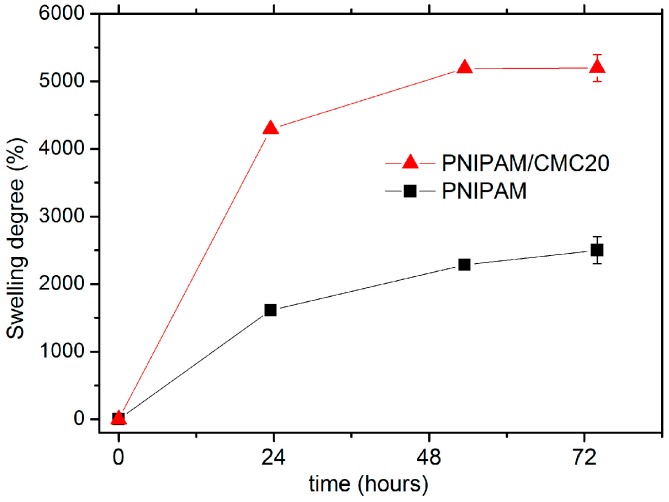
Swelling kinetics of PNIPAM and PNIPAM/CMC20 hydrogels in water at 295 K.

The attainment of the equilibrium swelling degree (SD_eq_) was checked by measuring swelling kinetics starting from lyophilized samples of the hydrogels (Figure 5). These data indicate that the swelling process is slightly faster for the PNIPAM/CMC20 hydrogel than for the pure PNIPAM hydrogel. The SD_eq_ measured at 295 K of the PNIPAM/CMC20 sample (5200% ± 200%) is much larger than that of the PNIPAM hydrogel (2500% ± 200%). On the contrary, the addition of 5 wt % of CMC to the PNIPAM hydrogels slightly reduces its SD_eq_ (1900% ± 100%). Similar SD_eq_ values (ca. 2000%) were measured for the PNIPAM/CMC5/Fe_3_O_4_ hydrogel. These results can be explained considering that the interactions of the hydrophilic groups of PNIPAM with those of CMC are more favorable than with water at low CMC concentration; thus, a decrease of the swelling degree is observed [15]. 

The VPTT estimated from the temperature at which the hydrogel samples start to be opalescent is not affected (within an uncertainty of ±2 K) by CMC. This result was confirmed by the swelling degree measurements as a function of temperature of PNIPAM/CMC20 hydrogels which show a drastic change from 303 to 307 K. Moreover, the presence of CMC/Fe_3_O_4_, at the concentrations used in this work, does not influence, within the accuracy of the measurements, the VPTT of the PNIPAM hydrogels. 

**Figure 6:**
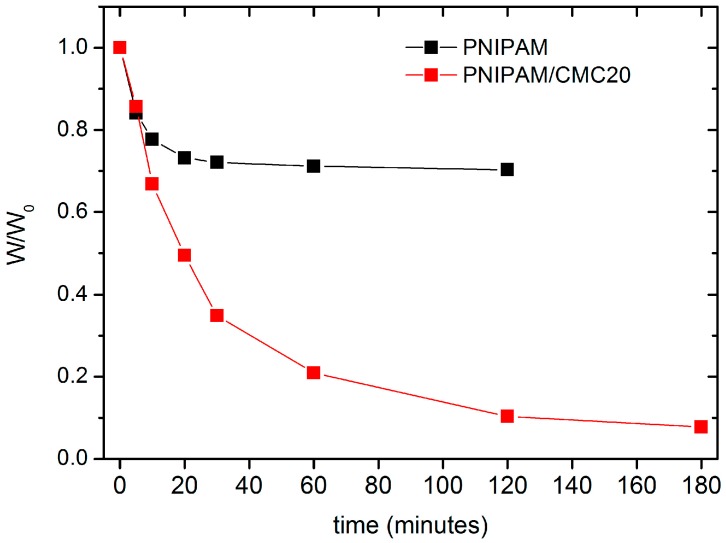
Water retention (defined as the weight W of the hydrogel divided by weight of the hydrogel swollen at 295 K W_0_) as a function of immersion time in water at 315 K for the pure PNIPAM and the PNIPAM/CMC20 hydrogels.

The deswelling kinetics of PNIPAM/CMC20 and of pure PNIPAM hydrogel samples, previously swollen in water at 295 K, were measured by monitoring the water retention % as a function of the immersion time in water at 313 K. After 30 min, the water retention of the PNIPAM/CMC20 hydrogel is reduced to about 35%, whereas that of pure the PNIPAM hydrogel reaches a steady-state value of 70% (Figure 6). Similar results were reported for PNIPAM/sodium alginate semi-IPNs [13]. The higher water retention of the pure PNIPAM hydrogel is due to the formation of a compact, vitreous layer at the surface of the samples above the VPTT, which hampers the release of water. The addition of CMC prevents the formation of this impermeable layer. This interpretation is supported by the different macroscopic morphology of the two kinds of hydrogels in water at 40 °C. The bubbles (full of water) that form at the surface of pure PNIPAM hydrogels in water above the VPTT [7] are not observed for the PNIPAM/CMC samples. 

**Figure 7:**
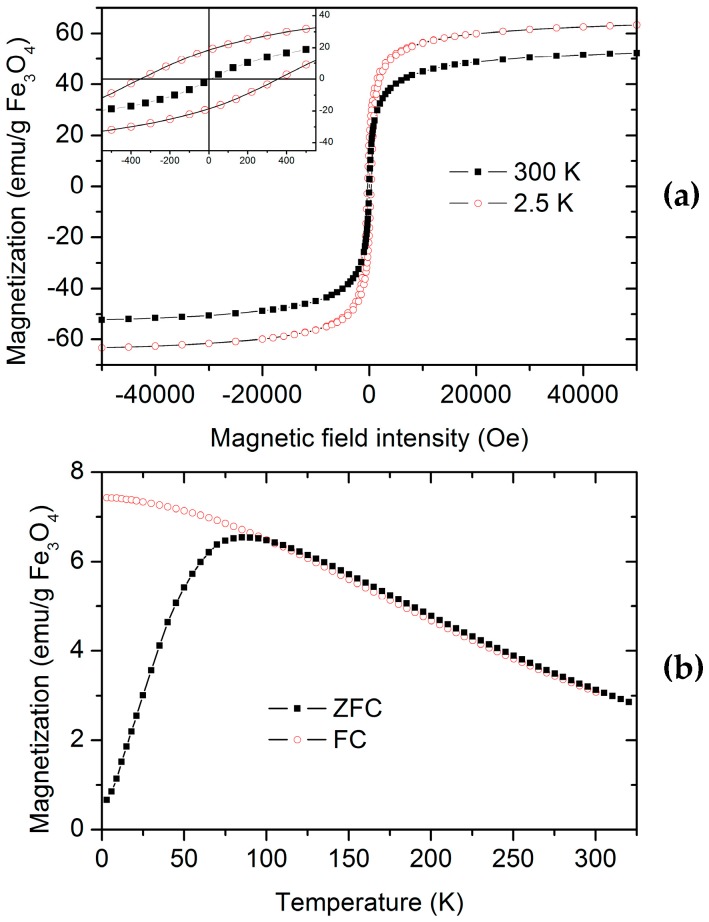
(**a**) Magnetization versus field intensity measured for the PNIPAM/CMC5/Fe_3_O_4_ lyophilized hydrogel at 2.5 K and 300 K. In the inset the curves at low intensity magnetic field are shown; (**b**) panel: zero field cooling (ZFC) and field cooling (FC) curves measured for the PNIPAM/CMC5/Fe_3_O_4_ sample. These curves were measured with an applied field of 50 Oe.

Magnetization vs. magnetic field intensity curves were measured for PNIPAM/CMC5/Fe_3_O_4_ and PNIPAM/Fe_3_O_4_ hydrogels to investigate the effect of CMC on the magnetic properties of the hybrid organic–inorganic hydrogels. Magnetization vs. magnetic field intensity curves of PNIPAM/CMC5/Fe_3_O_4_ and PNIPAM/Fe_3_O_4_ hydrogels show that the magnetic NPs are superparamagnetic. The magnetization vs. magnetic field intensity curves measured at 2.5 and 300 K for the PNIPAM/CMC5/Fe_3_O_4_ hydrogel are shown in Figure 7a. The curves measured at room temperature do not show any remanence. On the contrary, a hysteresis loop is observed in the curves measured at 2.5 K, with a remanent magnetization of 18 emu/g Fe_3_O_4_ and a coercive field of ca. 350 Oe (Figure 7a). The saturation magnetizations (63 and 52 emu/g Fe_3_O_4_ at 2.5 and 300 K, respectively) measured for the PNIPAM/CMC5/Fe_3_O_4_ hydrogel are higher than those of PNIPAM/Fe_3_O_4_ (46 and 33 emu/gFe_3_O_4_ at 2.5 and 300 K, respectively). These values are lower than that of bulk magnetite (92 emu/g at room temperature) but comparable with those of magnetite NPs of similar size [16]. The ZF and ZFC curves confirm that magnetite NPs in the PNIPAM/CMC5/Fe_3_O_4_ hydrogel are superparamagnetic with a blocking temperature (T_b_) of 83 K (Figure 7b). T_b_ measured for the PNIPAM/Fe_3_O_4_ hydrogel is 104 K, higher than that of the PNIPAM/CMC5/Fe_3_O_4_ hydrogel. Although many parameters can affect T_b_ (particle size, degree of oxidation, etc.), an increase in the number of particles in the clusters enhances the dipole–dipole interactions leading to an increase of T_b_ [16]. Hence, the observed difference of T_b_ suggests that the clusters of magnetite NPs are smaller in PNIPAM/CMC/Fe_3_O_4_ hydrogels than in PNIPAM/Fe_3_O_4_ hydrogels. 

## 3. Conclusions

The surface morphology at micrometric scale of PNIPAM/CMC hydrogels is characterized by a porous network structure which is not observed for pure PNIPAM hydrogels. These features are probably the emergence of micropores at the surface of the material. With an equal cross-linking degree, PNIPAM hydrogels containing 20 wt % of CMC have a much larger equilibrium swelling degree than pure PNIPAM hydrogels. The observed reduction of water retention for the PNIPAM/CMC hydrogels above the VPTT compared to pure PNIPAM hydrogels can reasonably be attributed to their surface morphology. The microporosity of the PNIPAM/CMC hydrogels prevents the formation of the dense layer impermeable to water as occurs for pure PNIPAM hydrogels. Moreover, the surface morphology of the PNIPAM/CMC5/Fe_3_O_4_ hydrogel is characterized by a honeycomb-like structure similar to PNIPAM/CMC hydrogels. Fe_3_O_4_ NPs prepared by co-precipitation in the presence of CMC embedded in PNIPAM hydrogels exhibit a superparamagnetic behavior. The lower blocking temperature measured for the PNIPAM/CMC5/Fe_3_O_4_ hydrogel compared to the PNIPAM/Fe_3_O_4_ hydrogel suggests that smaller aggregates of NPs are present in the PNIPAM/CMC5/Fe_3_O_4_ hydrogel. The capability of the PNIPAM/CMC hydrogels to absorb and release larger volumes of water at higher rates compared to pure PNIPAM hydrogels is important for many applications.

## 4. Materials and Methods 

NIPAM, *N*,*N*′-methylenebisacrylamide (BIS), *N*,*N*,*N*′*N*′-tetramethylenediamine (TEMED), K_2_S_2_O_8_, CMC sodium salt (molecular weight 700 kDa, 0.8 substitution degree), FeCl_3_∙6H_2_O, and FeSO_4_·(NH_4_)_2_SO_4_·6H_2_O were purchased from Sigma-Aldrich (St. Louis, MO, USA) and used as received. Deionized water was used for the experiments. PNIPAM hydrogels were prepared according to a free-radical polymerization procedure. The molar ratios of BIS, TEMED, and K_2_S_2_O_8_ with respect to NIPAM were 2%, 10%, and 0.25%, respectively. For the preparation of PNIPAM/CMC hydrogels, the reaction was carried out in water solutions of CMC. Hydrogels containing 5 and 20 wt % CMC with respect to NIPAM are indicated as PNIPAM/CMC5 and PNIPAM/CMC20, respectively. Fe_3_O_4_ NPs were prepared by co-precipitation from Fe(II)/Fe(III) (1:2 stoichiometric ratio) solutions by adding NaOH in the presence of CMC at 333 K [14]. The size of the Fe_3_O_4_/CMC NPs was 9 ± 1 nm, as determined by *X*-ray diffraction in a previous work [14]. The composition of the CMC/magnetite dispersions was 50 wt %. The preparation of the PNIPAM hydrogels containing 5 wt % CMC and 5 wt % Fe_3_O_4_ (with respect to PNIPAM) was carried out in aqueous dispersions of CMC/Fe_3_O_4_ NPs. The PNIPAM sample with embedded Fe_3_O_4_ NPs was prepared by carrying out the PNIPAM hydrogel synthesis in a water dispersion of Fe_3_O_4_ NPs, previously prepared using the coprecipitation method. The concentrations of magnetite in the samples (as determined by UV-visible spectrophotometry [14]) was 4.3 ± 0.2 wt % and 1.5 ± 0.2 wt % (with respect to PNIPAM) for the PNIPAM/CMC/Fe_3_O_4_ and PNIPAM/Fe_3_O_4_ samples, respectively. These samples will be indicated as PNIPAM/CMC5/Fe_3_O_4_ and PNIPAM/Fe_3_O_4_. After prolonged washing in deionized water to remove unreacted species, the hydrogels were frozen and lyophilized. 

The equilibrium swelling degree (SD_eq_), defined as (w − w_d_)/w_d_ %, where w and w_d_ are the weights of the swollen and of the dried hydrogel, was measured in deionized water at 295 K. Attenuated total reflectance–Fourier transform infrared (ATR-FTIR) spectra were collected using a FTS6000 spectrometer (Bio-Rad, Cambridge, MA, USA) at a resolution of 4 cm^−1^. A sigma VP FESEM microscope (Zeiss, Germany) was used for the FESEM measurements. The energy of the electron beam was in the range 1–10 keV, and the “in lens” detector was used to collect the secondary electrons. Measurements were performed under high vacuum conditions on the lyophilized samples without metallization or graphitization. 

Magnetization versus field intensity and zero field cooling (ZFC) and field cooling (FC) curves were measured by means of a Superconducting Quantum Interference Device magnetometer (Quantum Design Ltd. San Diego, CA, USA).
